# Similar Relative Carbon Costs for Construction and Storage of Sun and Shade Branches in Mature Temperate Trees

**DOI:** 10.1111/pce.70413

**Published:** 2026-03-04

**Authors:** Cedric Zahnd, Miro Zehnder, Ansgar Kahmen, Günter Hoch

**Affiliations:** ^1^ School of Biological Sciences University of Utah Salt Lake City Utah USA; ^2^ Department of Environmental Sciences – Botany University of Basel Basel Switzerland

## Abstract

Irradiance strongly affects the morphology, carbon (C) uptake and construction costs of leaves and branches. Within tree crowns, light decreases from the top downwards, but whether this translates to differences in the C balance of sun and shade branches remains unclear. Here, we combined a light‐driven photosynthesis model, parameterised with empirical data, with functional growth analyses to estimate the C costs and amortisation times of upper, sun exposed and lower, shaded branches in the crowns of mature trees from nine European species in a diverse and relatively open mixed forest. Amortisation times for the C costs of 1‐year‐old branches varied among species, but not between sun and shade branches except for two species. Expressed as percentage of the branch C uptake, branch costs were similar between crown positions in most species. Finally, a similar proportion of C assimilation is used for the seasonal build‐up of starch in upper and lower branches. Our results show that, at least in forests with relatively open canopies as the one studied here, the balance of assimilation and structural and non‐structural C costs at the branch‐level is finely tuned along the light gradient, suggesting a high degree of C autonomy even in shaded branches.

## Summary statement

Amortisation times and relative carbon (C) costs were similar in sun exposed and shaded branches of mature trees in a relatively open forest. The balance of assimilation and C costs at the branch‐level is finely tuned along the vertical light gradient, suggesting a high degree of C autonomy even in the most shaded branches.

## 1 Introduction

Light is a central, and highly variable resource for plants, setting an upper limit to the amount of carbon (C) they can assimilate. Within the crowns of large trees, light availability decreases exponentially from the top of the crown downwards as a function of the leaf area per unit ground area (Eliáš et al. 1989; Koike et al. 2004; Hertel et al. 2011). The light fraction reaching the lowest shade leaves of mature trees varies depending on the canopy structure, but typically ranges between 5% and 40% of the light above the canopy (Hollinger 1996; Koike et al. 2001; Bachofen et al. 2020; Zahnd et al. 2023). However, whether this gradient in energy input translates into a difference in the C balance, that is, the ratio of carbon cost to lifetime carbon uptake, between upper, sun exposed and lower, shaded branches depends on the adjustments to both the leaf gas‐exchange and the constructions costs within tree crowns.

To optimise whole‐crown C uptake, trees adjust a range of leaf traits along the vertical light gradient (Boardman 1977; Niinemets 1997; Lewis et al. 2000; Koike et al. 2001, 2004). These include specific leaf area (SLA; Gutschick and Wiegel 1988; Hollinger 1996; Poorter et al. 2009), chlorophyll and nitrogen concentrations (Ellsworth and Reich 1993; Niinemets et al. 1998; Koike et al. 2001), leaf size and display angle (Niinemets 1998; Hagemeier and Leuschner 2019) and leaf phenology (Yoshimura 2013; Gressler et al. 2015). All these adjustments contribute towards narrowing the difference in lifetime carbon uptake between sun and shade leaves. However, leaf construction costs (including structural and non‐structural carbon and the respiration costs associated with their biosynthesis) also vary along the same light gradient. To withstand increased water stress and excess irradiance, sun leaves have, for example, higher concentrations of lignin, alkaloids and phenolics compared to shade leaves (Li et al. 1993; Niinemets et al. 1998; Close et al. 2003; Poorter et al. 2006; Wilhelm and Selmar 2011; Cavatte et al. 2012). These compounds are metabolically expensive (Chung and Barnes 1977; Mooney and Gulmon 1982), and result in a positive correlation of leaf construction costs with irradiance in a wide range of study systems (Long 1934; Steubing et al. 1979; Sims and Pearcy 1991; Niinemets 1999; Baruch et al. 2000).

To date, only a few studies have simultaneously quantified construction costs and C uptake in mature tree canopies to specifically compare the amortisation times (i.e. the number of days a leaf or a branch has to assimilate C in order to amortise, or pay back, its construction costs) between sun and shade leaves or branches. Across both woody and herbaceous plants, lower light availability resulted in longer amortisation times among individuals growing in contrasting habitats (Williams et al. 1989; Poorter et al. 2006; Zhu et al. 2016) and in shading experiments (Poorter et al. 2006; Cavatte et al. 2012). To our knowledge, only Poorter et al. (2006) has compared sun and shade leaves of individual mature trees before. Across several temperate species, they found up to three times longer amortisation times in shade compared to sun leaves suggesting a considerably higher relative C investment per unit leaf biomass in the shade at the level of individual leaves.

Focusing on leaves only, these studies did not look at the costs of supporting woody structures. It has long been recognised that different strategies of investing in leaves and supporting woody structures have important implications for the C economy of trees (Corner 1949; White 1983; Niinemets et al. 2006). Indeed, tight scaling relationships between leaf area and either twig mass (Yang et al. 2009; Yang et al. 2010; Sun et al. 2019) or xylem area (Westoby and Wright 2003; Pickup et al. 2005; Lusk et al. 2007; Yang et al. 2014) reflect the trade‐offs between optimising light interception, mechanical stability, water supply and construction costs. Such trade‐offs have been explored among species differing in shade tolerance (White 1983), but, to our knowledge, so far not within individuals. Compared to lower branches, uppermost branches require more physical strength because of their more vertical growth and increased wind exposure (Eliáš et al. 1989; Daudet et al. 1999), and potentially more xylem area per leaf area due to higher hydrostatic pressures and increased evaporative demand (Burgess et al. 2006; Sellin and Kupper 2006; Zach et al. 2010). Accordingly, the ratio of leaf to supporting branch biomass might be a crucial parameter determining the branch C source‐sink balance along vertical canopy light gradients.

Besides structural C, trees also maintain non‐structural C reserves, mostly starch and low‐molecular sugars (non‐structural carbohydrates, NSC; Hoch et al. 2003). Particularly in young branches, starch concentrations show strong fluctuations during the growing season due to, among other factors, temporal imbalances of C source and sink strength (Hoch et al. 2003; Schadel et al. 2009; Klein et al. 2016). Recent studies found these temporal dynamics, as well as overall starch concentrations, to be very similar between upper, sun exposed and lower, shaded branches of mature trees (Schoonmaker et al. 2021; Zahnd et al. 2024), which may indicate a prioritised investment of C in storage over other C sinks like growth (Lacointe et al. 2004; Weber et al. 2019). However, similar starch dynamics and concentrations might also arise passively if trait adjustments to light are sufficient to equalise the overall source‐sink balance of leaves and branches within the canopy. In order to draw a definitive conclusion, analyses of the C balance at the whole branch level along the canopy light gradient are necessary.

In this study, we combined a light‐dependent photosynthesis model with branch‐level functional growth analyses to estimate the C costs and amortisation times of upper, sun exposed and lower, shaded branches in mature tree crowns. Including nine common temperate species with different leaf habits (deciduous broadleaved and evergreen coniferous trees) and wood anatomies (conifer, diffuse‐ and ring‐porous wood) allowed us to draw broad conclusions on how shade acclimations impact the branch‐level C balance. Based on previous studies, we expected physiological and morphological adjustments to partially, but not completely, compensate for the vertical canopy light gradient. Accordingly, we hypothesise that upper, sun exposed leaves and branches have shorter amortisation times and lower relative C investment in leaf and branch biomass compared to lower, shaded leaves and branches. Finally, we elaborated on these C balances by quantifying the costs associated with the seasonal build‐up of starch reserves in branches. We hypothesised that to reach similar starch tissue concentrations, lower, shaded branches have to invest a larger fraction of their total assimilates in C storage compared to upper, sun‐exposed branches.

## 2 Material and Methods

### 2.1 Site Description

The study was conducted at the Swiss Canopy Crane II site, located in the Jura foothills south‐east of Basel, Switzerland (47.439 N, 7.776 E, 550 m.a.s.l.). Average annual temperature and precipitation (1991 to 2020) at the site are 9.6°C and 972 mm, respectively, based on data from the nearby MeteoSwiss climate station in Rünenberg. The site comprises 1.68 ha of mature, temperate mixed forest with a total of 458 mature trees (diameter at breast height (DBH) ≥ 10 cm). European beech and Norway spruce are the most dominant of the 14 species growing at the site. The upper forest canopy reaches around 30 m tall but is heterogenous and relatively open (average leaf area index, LAI, of 2.2). A 50 m tall canopy crane with a 62.5 m jib is installed at the centre of the site, allowing direct access to the forest canopy via a manned gondola.

### 2.2 Sampling Design

In total, 32 mature and healthy trees (*N* = 2–5 per species depending on availability) from nine tree species were chosen for this study, including three evergreen conifer species (*Abies alba* Mill. [*N* = 4], *Picea abies* Karst. [*N* = 5], *Pinus sylvestris* L. [*N* = 4]) and six broadleaved angiosperm species (*Acer pseudoplatanus* L. [*N* = 3], *Sorbus torminalis* Crantz [*N* = 2], *Carpinus betulus* L. [*N* = 2], *Fagus sylvatica* L. [*N* = 4], *Quercus petraea x robur* [*N* = 5] and *Fraxinus excelsior* L. [*N* = 3]). The *Quercus* trees at the site are to varying degrees hybrids between *Q. petraea* Liebl. and *Q. robur* L. (Guggerli, unpublished data) but treated as one species here. For brevity, species will be referred to by their genus name hereafter. In each tree, two branches of ca 3 cm diameter were permanently marked, one in the uppermost crown and one in the lowest, most shaded part of the crown. Branches were all positioned on the outer surface of the tree crown, with their branching order depending on the species' crown architecture: In broadleaved species and *Pinus*, selected branches were several branching orders away from the trunk, while in *Picea* and *Abies*, we selected first‐ or second‐order branches. All measurements were taken either directly on these branches (non‐destructive sampling) or on close‐by branches (destructive sampling). To calculate the relative C costs of upper, sun exposed and lower, shaded branches, we conducted functional growth measurements and assessed tissue C concentration. Additionally, we used previously published data on leaf phenology, light availability, photosynthetic light response curves and seasonal dynamics of tissue starch concentrations, which were all measured on the same trees and using a similar study setup (Zahnd et al. 2023, 2024).

### 2.3 Branch Sampling and Functional Growth Analyses

Branches for both the chemical analyses and the functional growth measurements were sampled on the 30th of September 2021. By this time, annual branch growth was completed, but autumn leaf shedding had not yet begun. Two ca 5‐year‐old branches were collected in close proximity to the upper and lower permanently marked branches in each tree. On the first branch, leaves or 1‐year‐old needles and a 3‐ to 5‐year‐old wood segment were separated, immediately heat‐shocked in a microwave and dried at 80°C for 48 h. Samples were ground to a fine powder using a mixer mill (MM 400, Retsch GmbH, Haan, Germany) and then stored sealed over silica gel until chemical analysis was performed. The second branch was used for the functional growth analysis. All current‐year branch segments (*N* = 2–10) were separated from the branch. On each of those, one sided, projected leaf area (LA) was measured using a planimeter (LI‐3100C Area Metre, LI‐COR Biosciences GmbH, Bad Homburg, Germany) for broadleaved species and a flat‐bed scanner and dedicated digital image analysis tool (github. com/dabasler/LeafAreaExtraction) for conifer needles. The leaves and twigs (including wood and bark) were dried at 80°C for 48 h before all samples were weighed. For the conifers, the same procedure was additionally done with 1‐year‐old branch segments, needle dry mass and area of these were used only for calculating the costs of seasonal starch build‐up (see section ‘Relative C costs for seasonal starch build‐up’ below). All values from individual branch segments were considered technical replicates and averaged before further analysis.

### 2.4 Carbon Content Analysis

For analysing the carbon (C) fraction of wood and leaves, 1.3–1.6 mg of the ground leaf and wood samples were weighed into tin capsules (OEA Labs Ltd, Cornwall, UK). The samples were analysed with a Flash 2000 elemental analyser coupled to a DELTA Plus XP continuous‐flow IRMS via a ConFlo IV interface (Thermo Fisher Scientific, Bremen, Germany), and C concentrations are reported as % C per total dry mass. The measurements were conducted at the Stable Isotope Ecology Laboratory, Department of Environmental Sciences of the University of Basel, Switzerland.

### 2.5 Leaf Phenology and Light Measurements

The leaf phenology and light data we used here were previously described in detail by Zahnd et al. (2023). In brief, leaf development in spring, from budbreak to fully unfolded leaves, and leaf senescence in autumn (broadleaved only) were recorded one to two times per week on all the permanently marked branches. For the present study, the growing season was defined as starting when 50% budbreak was reached (all species) and ending at 50% leaf discoloration (broadleaved). For the purpose of this study, we did not define a growing season end for evergreen conifers, instead allowing photosynthesis and respiration time series to continue into the next year, as amortisation times for these species frequently exceeded a single year (see section *‘carbon assimilation model’* below. Note that temperature and light dependencies result in the expected negative net assimilation rates over winter). Continuous light measurements at 15‐min intervals were taken using automated light sensors (HOBO Pendant UA‐002‐64, Onset Computer Corp., Bourne Massachusetts, USA) on upper and/or lower permanently marked branches in a subset of the study trees. For the current study, we used measurements from 31 sensors throughout the year 2021. Measurements were transformed from lux (as measured by the sensors) to photosynthetically active photon flux density (PPFD, μmol m^−2^ s^−1^) using the empirically established relationship PPFD = lux/120 for this specific sensor type (Möhl et al. 2020).

### 2.6 Photosynthetic Light Response Curves (LRC)

Two photosynthetic light response curves (LRC) per species and crown position were measured in June and July 2021 using a LI‐6800 Portable Photosynthesis System (LI‐COR Biosciences GmbH, Bad Homburg, Germany). Chamber climate was set to 25°C, 60% relative humidity and saturating light (1500 µmol photons m^2^s^−1^, 70% red and 30% blue). Environmental conditions on the measurement dates were close to ideal for photosynthesis, with average midday temperature and relative humidity around 22°C and 57%, respectively, and mean soil moisture of 42% in a clay‐rich leptosol. LRCs were individually fitted with an exponential based model (Webb et al. 1974)

(1) $$P_{N}=P_{\mathrm{gmax}}\times \left(1-e^{\frac{-\phi_{\left(I_{0}\right)}\times I}{P_{\mathrm{gmax}}}}\right)-R_{d},$$

where *P*_*N*_ is the measured net photosynthesis rate [µmol CO_2_ m^2^s^−1^], *I* is the measured photosynthetic photon flux density [µmol photons m^2^s^−1^], *P*_*gmax*_ is the fitted maximum gross assimilation rate [µmol CO_2_ m^2^s^−1^], *R*_*d*_ is the fitted dark respiration rate [µmol CO_2_ m^2^s^−1^] and $\phi_{\left(I_{0}\right)}$ is the fitted quantum yield at *I* = 0 [µmol CO_2_ µmol photon^−1^].

### 2.7 Carbon Assimilation Model

Seasonal C assimilation was calculated based on photosynthetic light response curves (LRC) measured under near‐ideal conditions (i.e. no water limitation) and the continuous *in‐situ* light measurements along canopy gradients. To represent the seasonal course of photosynthesis and leaf respiration a temperature correction was applied (see below). In detail, we calculated 15‐min resolution time‐series of gross photosynthesis (*P*_*g*_) and dark respiration (*R*_*d*_) from the fitted LRCs and light recordings. Each LRC was used to calculate *P*_*g*_ and *R*_*d*_ based on each light logger from the same species and crown position (top or bottom), resulting in two to eight individual *P*_*g*_ and *R*_*d*_ timeseries per species and crown position (2 LRCs × 1 to 4 light loggers). As we had no loggers installed in *Sorbus* and *Acer* we used light data from the species with the most similar crown architecture and position within the forest canopy (*Carpinus* and *Quercus*, respectively), for these two species.

Hourly temperature data, measured at the closest MeteoSwiss climate station in Rünenberg, was used to correct the *P*_*g*_ and *R*_*d*_ timeseries. Following the approach from Kramer (1995), a multiplier function was applied to *P*_*g*_ assuming a broad optimum for photosynthesis from 10°C to 30°C, and a linear decline from there, reaching zero photosynthesis at −2 and +42°C, respectively. *R*_*D*_ was separately corrected using a Q_10_ temperature coefficient of 2. These temperature dependencies of photosynthesis and respiration correspond broadly to the typical values reported in Larcher (2003, 120–122) for the species studied here, but using different temperature responses did not change our overall findings (see Supporting Information Figure S1). We used the same temperature to correct values of upper, sun exposed and lower, shaded branches, since vertical temperature gradients are negligibly small for most of the season (Zahnd et al. 2023). After temperature correction, net assimilation was calculated from *P*_*g*_ and *R*_*d*_, which was then summed up over 24 h and multiplied by the molecular weight of C ( ~ 12), resulting in daily net C assimilation (*A*_*N*_, [grams C m^2^d^−1^]). Finally, the individual timeseries were averaged by species and crown position, resulting in 18 timeseries of mean *A*_*N*_ (9 species × 2 crown positions). These were used as the basis for all further calculations.

### 2.8 Carbon Costs and Amortisation Times

There are various methods for calculating construction costs, which take different aspects of structural, non‐structural and respirational costs into account (Vertregt and Penning de Vries 1987; Williams et al. 1987; Griffin 1994; Poorter 1994), but in general, construction costs are largely proportional to C content across major plant tissue compounds (Vertregt and Penning de Vries 1987; Poorter 1994). For the present study, we therefore accounted simply for the total C in the dry biomass of current‐year branches (called ‘carbon cost’ hereafter). This carbon cost underestimates the actual construction cost of tissues, as it does not account for respiration costs associated with the biosynthesis of the various components. However, given the tight correlation between carbon content and construction costs across major plant tissue compounds (Vertregt and Penning de Vries 1987; Poorter 1994) our bulk C concentration based estimate of carbon costs should accurately reflect differences in costs between upper, sun exposed and lower, shaded branches. Tissue C concentration was used to calculate the total C content in the current‐year twigs (wood + bark, *C*_*T*_, [g C branch^−1^]) and foliage (*C*_*F*_, [g C branch^−1^]).

For each branch, a timeseries of daily branch‐level C assimilation (*A*_*Branch*_) was calculated by multiplying the total branch leaf area with the average timeseries of daily *A*_*N*_ from the respective species and crown position. Two approaches were used to relate foliage and branch carbon costs (*C*_*F*_, *C*_*T*_) to their respective C uptake (*A*_*Branch*_): Firstly, the amortisation time of leaf and total branch costs were defined as the number of days it takes a branch to assimilate the equivalent amount of C invested in leaf (*C*_*F*_) and total branch (*C*_*F*_ + *C*_*T*_) biomass, respectively. Starting at 50% budbreak, daily *A*_*Branch*_ was cumulated until *C*_*F*_, and then *C*_*F*_ + *C*_*T*_ were reached. Whenever conifer amortisation times exceeded 31st December, the *A*_*Branch*_ time‐series was allowed to loop back to 1st January and continue from there. Secondly, *C*_*F*_ and *C*_*T*_ were expressed as percentage of the cumulative branch assimilation over the whole lifespan of the foliage (relative carbon cost; RCC). Being winter‐deciduous, the leaf‐lifespan of broadleaves species was the period from 50% budbreak in spring to 50% leaf discoloration in autumn. For the conifers, an average 5‐year leaf‐lifespan was assumed for *Picea* and *Abies*, and 3 years for *Pinus*, based on personal observations of retained needle generations and in accordance with estimates from Leuschner and Meier (2018).

### 2.9 Sensitivity Analysis

To test the effects of different traits on the relative C costs in lower, shaded branches, we conducted a simple sensitivity analysis. We recalculated the whole‐branch (twig + foliage) RCC of lower, shaded branches while one by one replacing all relevant components of the calculation with values from the upper, sun exposed crown. The replaced factors were (1) incident light, (2) *P*_*gmax*_, (3) *R*_*D*_, (4) $\phi_{\left(I_{0}\right)}$, (5) specific leaf area (SLA), (6) the ratio of leaf area to branch dry weight (LA:BDW), (7) tissue C concentration and (8) season length. For each factor replacement, the relative change in RCC was calculated by dividing the difference between new and initial RCC by the initial RCC.

### 2.10 Relative C Costs for Seasonal Starch Build‐Up

To calculate the relative C investment of young branches in the seasonal build‐up of local starch reserves, we used the *A*_*Branch*_ time‐series described above along with seasonal dynamics of branch starch concentrations from Zahnd et al. (2024). The starch dynamics were measured in the same trees as this study, but in the year 2020. Based on point measurements of starch from 2019 to 2021 however, the magnitude of seasonal starch dynamics are likely similar across years (Zahnd et al. 2024). We calculated the absolute amount of starch, in grams per branch, being built up by multiplying the amplitude of starch concentration (see Figure 3a) by the respective tissue dry mass. For conifers, the needles on 1‐year‐old branch segments were used. For the broadleaved species, starch had been measured in 3–5‐year‐old wood, but the leaf area and biomass were only available from current year branches (see above). Since we did not have data on the branching structure in upper, sun exposed and lower, shaded branches, and to preserve our measured ratio of leaf‐ to twig biomass between crown positions, we estimated the fraction of a 5‐year‐old branch which would be supplied with C by the foliage of a single current‐year branch assuming that branch biomass increment is constant each year, and that every year, on average 2 new shoots grow on each previous one. Following these assumptions, the biomass of the current year branch was upscaled to a 5‐year‐old branch as:

(2) $${{\mathrm{Biomass}}_{5}=\mathrm{Biomass}}_{1}\times \sum_{i=1}^{5}\frac{i}{2^{i-1}}={\mathrm{Biomass}}_{1}\times 3.5625,$$

where *Biomass*_1_ is the current year dry mass (wood + bark), *i* the branch segment age (where 1 = current year), and the *i* fractions in the sum reflect the ratios of twig biomass to leaf area of increasingly older branch segments. The total amount of starch in the respective tissues was then converted to grams C by multiplying it by the C fraction of starch (0.4). Using the *A*_*Branch*_ timeseries, we calculated the relative C cost of local starch reserves by expressing the total starch C as a percentage of the branch net C uptake over the period of starch accumulation (see Figure 3a).

### 2.11 Statistical Analysis

All variables of interest were analysed with linear mixed effects models using crown position (top vs bottom), species and their interaction as fixed effects and the tree individual (tree ID) as random intercept to account for the paired nature of samples. Models were visually inspected for key assumptions, including normality, heteroscedasticity and outliers (Zuur et al. 2009). Where necessary, dependent variables were log‐transformed to ensure normality of residuals. Post‐hoc *t*‐tests were used to test differences between canopy positions in each species individually. All analyses were done using the R statistical environment (R Core Team 2025). The packages ‘lme4’ (Bates et al. 2015) and ‘lmerTest’ (Kuznetsova et al. 2017) were used for model fitting and testing. Post‐hoc tests were performed using the package ‘emmeans’ (Lenth 2022).

## 3 Results

### 3.1 Amortisation Times and Relative C Costs

**Figure 1:**
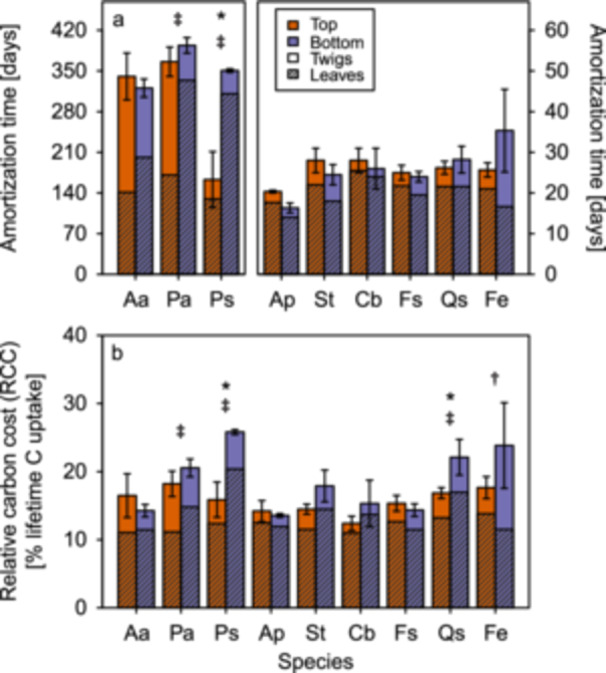
Amortisation time and relative carbon costs of current‐year branches in the upper (orange) and lower (purple) crown areas of the nine species. (a) Amortisation time for current year leaf (hatched) and twig (wood + bark; plain) carbon costs in days since budbreak. (b) Percentage of lifetime branch C uptake invested in leaf (hatched) and twig (wood + bark; plain) biomass. Standard error bars refer to the total values (leaf + twig). Species are shown on the x‐axis (Aa = *Abies*, Ap = *Acer*, Cb = *Carpinus*, Fs = *Fagus*, Fe = *Fraxinus*, Pa = *Picea*, Ps = *Pinus*, Qs = *Quercus*, St = *Sorbus*). Significant differences between crown positions are indicated with different symbols for leaves (**‡**), twigs (**†**) or total (*). Significances are based on post‐hoc t tests (*p* < 0.05). Note the different y axis scale for the three conifers in (a).

Across all species, whole‐branch amortisation times did not differ significantly between crown positions (Table 1). The calculated amortisation times of both leaf and total branch carbon costs (i.e. the number of days a leaf or branch has to assimilate C in order to amortise, or pay back, its carbon content) were much longer for conifers (up to 400 days) compared to broadleaved species (ca 20–30 days), but relatively similar within these groups (Figure 1a, Table 1). Leaf amortisation times were very similar between crown positions in most species, although significantly different across all species, with an additional position x species interaction (Table 1). Individually, only *Picea* and *Pinus* had significant differences in leaf amortisation times between the crown positions, with about twofold amortisation times for needles in the shade compared to those in the sun (Figure 1a, hatched bars). This resulted in significantly different whole‐branch (i.e. also considering the C costs for the branch wood production) amortisation times of lower, shaded branches in *Pinus*, but not in *Picea*, because of the much longer amortisation time of upper twigs (wood and bark) in the latter (Figure 1a).

**Table 1:** Summary of the type III ANOVA results (Satterthwaite's method). Percentage of the total sums of squares of crown position, species and the interaction effect are given with the respective significances (stars). Values in parentheses under the explanatory variable names are the respective degrees of freedom. (log) after the variable names indicates that the variable was log‐transformed for the analysis. For full ANOVA tables see Supporting Information Table S1.

Response	Crown pos. (df = 1)	Species (df = 8)	Crown pos. × Species (df = 8)
Leaf amortisation time (log)	0.30*	91.03***	4.02***
Total amortisation time (log)	0.09^ns^	77.90***	1.85**
Relative leaf C cost (log)	8.07**	20.86*	25.67**
Relative twig C cost (log)	1.02^ns^	15.03*	6.74^ns^
Total relative C cost (log)	6.86**	18.71*	14.20^ns^
Starch relative C cost (log)	0.42^ns^	43.13***	1.13^ns^

*** *p* ≤ 0.001

** *p* ≤ 0.01

* *p* ≤ 0.05; ns *p* > 0.05.

When expressed as a percentage of the total C uptake over the whole leaf‐lifetime, the leaf‐ and whole‐branch RCC differed significantly among species and crown positions (Table 1), but notably, differences were small both among species and between upper, sun exposed and lower, shaded branches, and values fell within a narrow range of on average 12%–25% (Figure 1b). This is despite the significantly higher per‐leaf‐area seasonal C uptake of upper, sun exposed branches in most species (25%–123% higher than shade branches; Supporting Information Figure S2, Table S2) and the overall significantly lower per‐branch C uptake of lower, shaded branches, although the latter was not uniformly so across species (Supporting Information Figure S2, Table S3). Individually, only *Picea*, *Pinus* and *Quercus* spent a significantly larger percentage of C assimilates on leaf biomass in lower branches, which resulted in significantly higher relative whole‐branch C costs of lower, shaded branches in the latter two species (Figure 1b). RCC for twigs (wood + bark) were very similar in upper, sun exposed and lower, shaded branches of all species but *Fraxinus*, and pronouncedly smaller than the RCC for leaves (Figure 1b, Table 1). Notably, although crown position effects were often significant when testing amortisation times and RCC across all species, they accounted only for a small fraction of the variation (< 10% of total sum of squares; Table 1).

### 3.2 Key Trait Adjustments Along the Canopy Light Gradient

**Figure 2:**
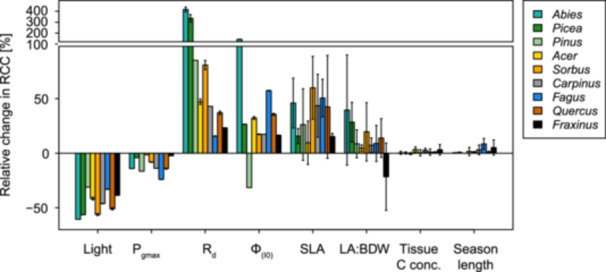
Sensitivity analysis for the RCC calculation of lower, shaded branches. Relative change (mean and 95% CI) in shade branches (leaf + twig) RCC when replacing different factors (x axis) with values from the top canopy. Colours indicate the different species. Note the broken y‐axis at 100% and the 10x reduced scale of the axis above 100%.

To test the importance of different traits for the branch C balance, we recalculated the RCC of lower, shaded branches while replacing key input variables of the calculation one by one with values from the upper canopy in a sensitivity analysis (Figure 2). RCC of shade branches were on average 46% (31%–61%, depending on species) lower when using the incident light from the top of the canopy (Figure 2). Among the photosynthetic LRC parameters, *P*_*gmax*_ had the smallest effect with on average 11% (1%–24%) lower RCC when using *P*_*gmax*_ from the upper canopy, whereas the parameter related to low‐light assimilation efficiency $[\phi_{\left(I_{0}\right)}$; 35% (17%–143%) higher RCC except *Pinus* with 32% lower RCC) and *R*_*d*_ (121% [16%–417%] higher RCC) had substantial effects in most species, particularly in the thick‐leaved conifers (Figure 2). This reflects the more consistent and stronger crown position differences in $\phi_{\left(I_{0}\right)}$ and *R*_*d*_ compared to *P*_*gmax*_ (Supporting Information Figure S3, Table S2). SLA had a strong effect in several, but not all species (34% [10%–60%] higher RCC). The ratio of leaf area to twig mass meanwhile tended to have smaller and less clear effects (95% CI overlapped with zero in most species), with the strongest effects in the two conifers *Abies* and *Picea* (12% [5%–40%] higher RCC except Fraxinus, with 22% lower RCC; Figure 2). SLA showed more consistent and stronger differences across the canopy compared to LA:BDW (Supporting Information Figure S4, Table S3). Finally, the tissue C concentration and the season length had negligibly small effects in all species (< 9%; Figure 2), since these values showed little or no difference between crown positions (Supporting Information Figure S5, Table S3 and Zahnd et al. 2023).

### 3.3 C Costs of Seasonal Starch Build‐Up

**Figure 3:**
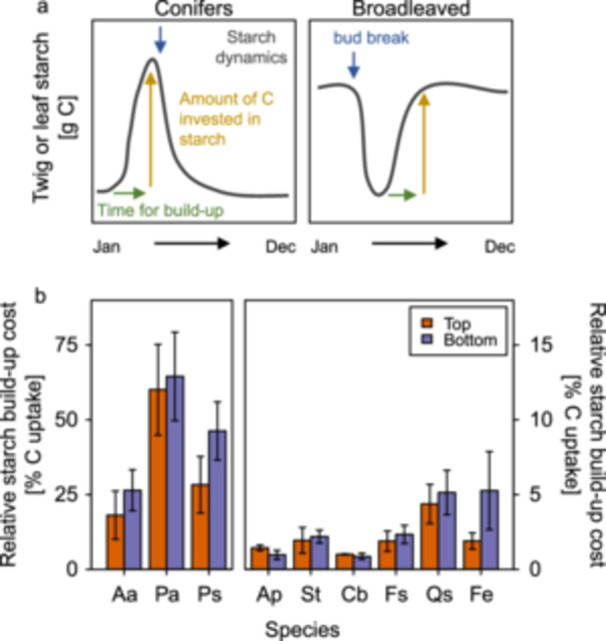
Relative C costs of the seasonal starch build‐up in 1 year old needles (conifers) and 5 year old branches. (a) Conceptual representation of the seasonal starch dynamics (grey curve) in 1 year old needles (conifers; left panel) and 5 year old branches (wood only, broadleaved species; right panel). Green arrows show the time of year when starch is built up, determining the period over which branch C uptake was summed for this calculation. Yellow arrows represent the amount of C invested in local starch reserves during that time. (b) C costs of the seasonal build‐up of local starch reserves in upper (orange) and lower (purple) branches, expressed as percentage of branch net C uptake during the period of starch build‐up. Species are shown on the x‐axis (Aa = *Abies*, Pa = *Picea*, Ps = *Pinus*, Ap = *Acer*, St = *Sorbus*, Cb = *Carpinus*, Fs = *Fagus*, Qs = *Quercus*, Fe = *Fraxinus*). Note the different y axis scale for the three conifers in (b).

Relative costs of seasonal starch build‐up varied significantly among species (Table 1). Conifers, which accumulate starch in mature needles in early spring before bud break (Figure 3a), invested 20%–65% of concurrent assimilation in local starch reserves (Figure 3b), while values were much lower (< 5%; Figure 3b) for broadleaved species, which accumulate starch in branch sapwood during the first half of the growing season following bud break (Figure 3a). None of the species had significantly different relative starch build‐up costs between upper, sun exposed and lower, shaded branches (Figure 3b, Table 1). Indeed, values were very similar between crown positions in most species, although, for the broadleaved species, this finding depends somewhat on the assumption that sun‐ and shade branches have similar branching patterns (i.e. number of lower order branches per higher order branch, see methods). As reported in Zahnd et al. (2024), the seasonal starch dynamics are very similar in upper, sun exposed and lower, shaded branches of most species. Accordingly, neither the seasonal amplitudes (minimum to maximum concentration) of starch concentrations, nor the duration of starch build‐up differed significantly between crown positions, with the exception of the larger amplitude in lower, shaded *Picea* needles (Supporting Information Figure S6b,d, Table S4). Since in most species also the twig or needle biomass was very similar between crown positions, the total C in local starch reserves did not differ significantly between upper, sun exposed and lower, shaded branches (Supporting Information Figure S6a,c, Table S4). The two ring‐porous species (*Quercus* and *Fraxinus*), however, were an exception: both twig biomass and starch amplitude tended to be higher in upper, sun exposed branches, resulting in much larger total starch C pools, although significantly so only in *Fraxinus* (Supporting Information Figure S6). Despite this, relative starch build‐up cost was similar between crown positions in *Quercus*, and only insignificantly higher in lower, shaded branches of *Fraxinus* (Figure 3b).

## 4 Discussion

In this study, we showed that the amortisation times for the C costs of new branches vary widely among species, but are similar in upper, sun exposed and lower, shaded branches of most species. Most interestingly however, relative carbon costs (RCC) for current‐year branches (leaves and twigs) turned out to be surprisingly similar between crown positions and species. Key traits adjusted in the shade included SLA, dark respiration rates and photosynthetic efficiency in low light, whereas maximum assimilation rates, tissue C content and season length played a minor role. We further showed that a similar proportion of the concurrent C assimilation is required for the seasonal starch build‐up in upper, sun exposed and lower, shaded branches. Our data suggest that the balance of assimilation and both structural and starch reserve C costs at the branch‐level is finely adjusted and surprisingly similar along the vertical light gradient within the tree crowns of the investigated, relatively open forest.

### 4.1 Minor Differences in Amortisation Times and Relative Carbon Costs

We found very similar amortisation times between sun and shade leaves in seven of the nine species studied here. This seemingly contradicts previous studies reporting that leaf amortisation times became longer with decreasing light availability along natural light gradients (Williams et al. 1989; Poorter et al. 2006) and under experimental shading (Poorter et al. 2006; Cavatte et al. 2012). In the, to our knowledge, only other study comparing amortisation times within individual mature trees, Poorter et al. (2006) found significantly longer amortisation times of shade compared to sun leaves. They reported particularly large differences for *Fagus* and *Carpinus*, with 15–20 days for sun leaves, but up to 75 days for shade leaves, which is over three times our estimates for shade leaves of these species. The most likely reason for these contrasting findings is the less severe light gradient in our study compared to previous studies. Due to the relatively open canopy at our study forest (the average LAI was 2.2), irradiance in the lower relative to upper leaves (relative irradiance, RI) of broadleaved species with tall crowns, such as *Fagus*, was ca. 30% during the growing season, with values as low as 15% in *Abies*. In comparison, Poorter et al. (2006) reported for a mixed forest close to our site lower RI at the canopy bottom of 5%–30%, with *Fagus* and *Carpinus*, which showed the strongest difference in amortisation times, at the lower end of these RI values. In combination, these results suggest that at a RI of ca 30% and above, leaf adjustments are sufficient to fully compensate the lower irradiance and C uptake, but C amortisation times start to increase at some threshold below 30% RI despite morphological and physiological adjustments. Notably though, leaves of both temperate trees and tropical shrubs were found to turn net C negative only at < 2% RI (Williams et al. 1989; Poorter et al. 2006). Our data thus suggest that even in denser forests than at our study site, the vast majority of the canopy foliage experiences light environments in the range we measured here, and thus has equal leaf C amortisation times and comparable levels of C autonomy, with only the lowermost branches experiencing insufficient light, increased amortisation times and potentially even a negative C balance.

In our study, we expanded the calculations of leaf amortisation times and RCC by including the costs of entire current‐year branches (leaves and twigs). While sun twigs had a much larger dry mass than shaded ones in some species, the resulting whole branch amortisation times were very similar between crown positions in all species except *Pinus*. This shows that similar to individual leaves, carbon costs at the whole‐branch level are generally also adjusted to the C uptake of a given branch along the canopy light gradient, at least within the range of light measured here. Total amortisation times for leaves plus twigs of a current‐year branch were 20–35 days in broadleaved species and up to 400 days in the evergreen conifers, substantially shorter than the foliage lifespans of 167–202 days for broadleaved species (Zahnd et al. 2023) and > 3 years (> 1000 days) in the conifers (Leuschner and Meier 2018). Accordingly, branches of all species were unequivocally highly positive in their C balance over their foliage lifetime irrespective of their crown position. This positive C balance may explain why we did not observe self‐pruning of shade branches in our forest. Indeed, a recent study in a much denser, young forest plantation found self‐pruning, i.e. the shedding of shaded branches presumably due to a negative C balance, occurs only at much lower RI than we observed, below ca 5%–0.25% depending on species and forest composition (Kothari et al. 2025).

It is well known that leaf construction costs and amortisation times both increase with increasing leaf lifespan (Chabot and Hicks 1982; Williams et al. 1989; Kikuzawa and Lechowicz 2011), a relationship that is reflected in the higher foliage and wood C content, and longer amortisation times of evergreen conifers compared to the deciduous broadleaved species in our study. To account for different leaf lifespans, we calculated the relative C cost (RCC), i.e. the percentage of lifetime C uptake which is invested in the current year branch tissues. We found a surprisingly narrow range of RCC of 10‐20% for leaves and needles and 5‐10% for twigs across both species and crown positions. With respect to the species comparison, our model might have somewhat underestimated the true variation in RCC by applying simplified assumptions in some traits (e.g., same temperature dependence of photosynthesis across species, rough estimates of mean conifer needle lifespan). However, we would not expect fundamental shifts in RCC among species even if more precise, species‐specific values for those traits had been used in our calculations. Overall, our study thus suggests an upper limit of around 25% for the RCC of branches across different light environments, species and leaf habits, with substantially higher RCC likely not being sustainable, as sufficient surplus photoassimilates are required for export to growth, respiration and other C sink activities in all other parts of the tree. However, explicitly accounting for these additional C costs requires a good understanding of how much upstream, non‐photosynthetic biomass each young branch supplies with C (see e.g. Witowski 1997), which remains difficult for large forest trees. Nevertheless, the similar and relatively low RCC of upper, sun exposed and lower, shaded branches indicate a high degree of C autonomy even in shade branches, in accordance with the branch autonomy theory (Sprugel, Hinckly & Schaap 1991), and similar proportions of assimilates being exported to the rest of the tree across the vertical canopy gradient. This may further suggest that the shade crown, rather than merely amortising itself, might contribute substantially to the whole‐tree C balance (Law et al. 2002; Chen et al. 2012). This is probably even more important than suggested by our simple calculations, since our model did not account for negative effects of high VPD on photosynthesis, which would restrict C‐assimilation significantly more in the sunlit upper crown parts compared to the lower, shaded branches (Law et al. 2002).

There were some exceptions to the general pattern. *Picea* and *Pinus* both had higher leaf amortisation times and RCC in shade compared to sun leaves. This may partially be because these species had only minor SLA adjustments, but could also be biased by our needle lifespan estimates. In evergreen conifers, needles on shade branches commonly have a longer lifespan than sun‐exposed ones (Kikuzawa and Lechowicz 2011), accounting for which may further reduce the difference in RCC we observed. Additionally, some differences may partially have arisen from measurement difficulties compounded by our low per‐species replication. For instance, two of the three species with clear differences in RCC between upper, sun exposed and lower, shaded branches have a peculiar response to $\phi_{\left(I_{0}\right)}$ (*Pinus*) and LA:BDW (*Fraxinus*) in our sensitivity analysis (Figure 2). These could be artefacts related to the difficulty of accurately measuring photosynthesis on *Pinus* needles and the atypical crown architecture of Fraxinus (few thick shoots with very large leaf area).

### 4.2 Key Trait Adjustments Along the Canopy Light Gradient

Amortisation times and RCC depend both on the total C uptake and the carbon cost of branches. In our study, the lower area‐based C uptake of shade leaves was predominantly caused by the lower light availability itself (Figure 2). Higher maximum assimilation rates (*P*_*gmax*_) in sun leaves would be expected from the literature (Koike et al. 2001; Larcher 2003; Poorter et al. 2006), but we observed this only in four of the nine studied species: the tall‐crowned *Fagus* and *Quercus*, and surprisingly also *Pinus* and *Sorbus*, both of which have particularly short crowns. As discussed above, *P*_*gmax*_ differences between sun and shade leaves might be larger in denser forests with stronger light gradients than our study site. Our sensitivity analysis indicated that indeed, *P*_*gmax*_ had a minor effect on RCC across all species, whereas the photosynthetic traits related to low‐light efficiency (Rd, $\phi_{\left(I_{0}\right)}$) had strong compensatory effects on RCC. Another key factor in compensating for lower C uptake in lower, shaded branches was the SLA, a central trait in plant adjustments to varying light (Evans and Poorter 2001; Poorter et al. 2006). Only the two conifers *Picea* and *Pinus* had almost no adjustment of SLA along the canopy gradient, which also explains why we observed much longer amortisation times of shade leaves in those two species. Interestingly, those were also the only species for which leaf area per branch dry weight (LA:BDW) had a substantial effect on RCC (Figure 2), with LA:BDW otherwise showing inconsistent patterns across species. Season length and tissue C concentration both had negligible effects on RCC in lower, shaded compared to upper, sun exposed branches. C content was similar between crown positions in the conifers, and only slightly higher in the sun leaves of some broadleaved species. Previous studies have also found that within tree crowns, leaf construction costs tend to increase with irradiance, but the differences between sun and shade leaves were usually small (Niinemets 1999; Poorter et al. 2006). In summary, we found shade adjustments broadly in agreement with previous studies, with adjustments to SLA and traits related to low‐light photosynthetic efficiency mostly sufficient for compensating the lower light availability in shade branches.

### 4.3 The Relative Cost of Seasonal Starch Build‐Up

We further elaborated on our analysis by looking at the cost of one key non‐structural carbon pool: the local starch reserves in branches. Comparing the amount of starch which is built up in young twigs (broadleaved) or mature needles (conifers) during a season to the total concurrent C uptake of those branches, we found very similar proportions of assimilates required for the seasonal starch build‐up in upper, sun exposed and lower, shaded branches within each species. Crucially however, the proportion of concurrent assimilates required for the seasonal starch build‐up was always less than 100%, suggesting that the starch formation in branches can principally occur autonomously at the branch‐level and without necessitating import of C from other parts of the tree. For evergreen conifer needles, the percentage C uptake required for starch build‐up was relatively high (up to 65% in *Picea*), but these species accumulate starch very fast in early spring prior to primary and secondary tissue growth, when little other C sinks are competing for assimilates (Schadel et al. 2009; Zahnd et al. 2024). In contrast, deciduous broadleaved species refill most of their starch reserves in the first half of the growing season, concurrently with the peak activity of cambial growth of stem and branches. However, studies found young branches become net C positive very quickly after budbreak (Keel and Schadel 2010; Landhäusser 2011). Given the small percentage of total concurrent C uptake required for the starch build‐up in these species (< 5%), and the relatively short tissue amortisation times, we conclude that current local assimilates are most likely sufficient for building up starch reserves even in the lowest crown positions. Indeed the low relative structural and non‐structural costs leave considerable room for other potential C sinks not considered here (e.g. storage lipids [Hoch et al. 2003], respiration costs associated with the biosynthesis of structural and non‐structural C compounds) without changing our conclusion. Furthermore, our findings agree with previous labelling experiments, which found high C autonomy of branches under most conditions (Sprugel et al. 1991; Lacointe et al. 2004; Volpe et al. 2008). Recently, surprisingly similar seasonal dynamics and concentrations of starch in branches along the vertical light gradient of tree crowns were reported (Schoonmaker et al. 2021; Zahnd et al. 2024). Previous deep shade experiments with tree saplings suggested that such similar starch concentrations despite stark light gradients indicate an ‘active’ C investment towards storage at the cost of other sink activities (e.g. growth; Lacointe et al. 2004; Weber et al. 2019). In mature trees, this would imply either that assimilates are redistributed from the upper to the lower branches, or that lower, shaded branches invest a larger proportion of assimilates in starch. However, our calculations instead suggest that a ‘passive’ model of C allocation can sufficiently explain the similar starch concentrations and dynamics along the canopy gradient, where these simply reflect the very similar net C‐balance of upper, sun exposed and lower, shaded branches.

### 4.4 Limitations of This Study

In calculating amortisation times and RCC, we made several simplified assumptions which may have affected our findings. Notably however, all assumptions were made so that we would, if anything, have underestimated amortisation times and RCC more in upper, sun exposed compared to lower, shaded branches. More detailed data or models would therefore most likely strengthen our finding that, contrary to our hypothesis, sun exposed branches of the upper part of mature tree crowns do not have shorter amortisation times and lower RCCs than branches from the shaded lower crown.

Firstly, by using bulk C content (carbon costs) as a proxy for construction costs, we disregarded the respiration costs associated with the initial biosynthesis of the tissues, and thus systematically underestimated relative construction costs. Respiration costs vary among tissue constituents but are on average around 25% of the accumulated carbon (Larcher 2003). Using more elaborate methods to estimate construction costs, previous studies found leaf and twig construction costs of 0.52–0.72 g C g^−1^ dry mass (Poorter 1994; Poorter and Villar 1997; Niinemets 1999), which is between 0% and 56% higher than our simpler estimates. Even accounting for the highest case, our RCC estimates would be on average 32% of the leaf lifetime C uptake, with the highest RCC still well below 100%. Furthermore, bulk C content was shown to correlate well with construction cost estimates including respiration costs (Vertregt and Penning de Vries 1987; Poorter 1994). We are therefore confident that, while we underestimate the absolute construction costs, the differences in construction costs between upper, sun exposed and lower, shaded branches are accurately reflected by bulk C content.

Secondly, we also used a relatively simple approach for calculating C uptake. While much more sophisticated photosynthesis models are available, using light and the photosynthetic response to it as the primary drivers of C uptake along a shading gradient seems reasonable and corresponds to approaches used in similar studies (Poorter et al. 2006). Accounting for additional factors, such as VPD, would likely limit stomatal conductance and photosynthesis more in sun‐exposed leaves than in shaded ones (Jifon and Syvertsen 2003; Niinemets et al. 2004; Bachofen et al. 2020), thus increasing their RCC more than that of lower, shaded canopy branches. Additionally, while diurnal and seasonal variation in light environments are captured well by our 15 min resolution light measurements, shorter‐term variation is not. Sunflecks, caused by for example, wind or moving clouds, are traditionally thought to be particularly important for photosynthesis in the understory and in shaded parts of the forest canopy, as they provide periods of high irradiance (Way and Pearcy 2012), although more recent studies indicate that sunflecks may play an important role even in upper canopy layers (Durand et al. 2022). How sunflecks affect the C balance of sun and shade branches represents an interesting avenue for future research, but for the current study, we consider it likely that sunflecks would, if anything, disproportionately benefit the C balance of lower, shaded compared to upper, sun exposed branches (Way and Pearcy 2012). Taken together, using more sophisticated approaches to our calculation would most likely lead to even smaller differences in RCC between upper and lower branches, or even to lower, shaded branches having a more favourable C cost‐benefit ratio than upper, sun‐exposed ones, thus clearly contradicting our initial hypothesis.

Finally, our analysis has a key limitation shared by the broader theory of branch autonomy (Sprugel, Hinckly & Schaap 1991). We only consider the carbon household; however branches use other resources supplied by roots and stems (water, nutrients), the availability of which in turn affects the ability to assimilate C (Sprugel 2002). Future assessments of relative branch costs should therefore take resources other than carbon into account too.

## 5 Conclusions

With this study, we showed that the amortisation times and relative construction costs are very similar for upper, sun exposed and lower, shaded branches and among tree species. This illustrates the well‐established capacity of trees to acclimate to the within‐crown light gradient, and shows that a difference in the cost‐benefit ratio of branches cannot be *a priori* assumed from a light gradient alone. However, with more severe shading, acclimations may become insufficient, resulting in longer amortisation times of shade leaves. For the temperate trees studied here, this appears to be the case for relative irradiance lower than 30%, although leaves remain net C positive in much darker environments (Poorter et al. 2006). It further follows that interpreting starch storage concentrations and dynamics along the vertical canopy gradient requires an understanding of the actual C balance of upper, sun exposed and lower, shaded branches. Finally, while our calculations were limited to individual branches, they indicate that the shade canopy may contribute significantly to the whole‐tree C balance, especially during dry periods in summer when photosynthesis is typically more limited in the upper canopy due to higher temperature and VPD (Law et al. 2002; Chen et al. 2012).

## Conflicts of Interest

The authors declare no conflicts of interest.

## Supporting information


**Fig. S1:** Relative carbon costs of current‐year branches when using different temperature corrections for photosynthesis and leaf respiration. **Fig. S2:** Seasonal C uptake in upper (plain) and lower (hashed) branches. **Fig. S3:** Fitted parameters from the photosynthetic light response curves in upper (plain) and lower (hashed) branches. **Fig. S4:** Specific leaf area (SLA; a) and leaf area to branch dry weight ratio (LA:BDW; b) in upper (plain) and lower (hashed) branches. Significances are based on post‐hoc t tests (* *P* < 0.05). **Fig. S5:** Twig (a) and foliage (b) tissue C concentration in upper (light grey) and lower (dark grey) branches. Significances are based on post‐hoc t tests (* *P* < 0.05). **Fig. S6:** Parameters used for the calculation of relative starch build‐up costs in upper (plain) and lower (hashed) branches. **Table S1:** Full type III ANOVA results (Satterthwaite's method), completing main text Table 1. **Table S2:** Type II ANOVA results for those parameters involved in the amortization time and RCC calculations for which the N was not sufficient to include a tree ID as random effect (Per‐area C uptake and fitted LRC parameters). **Table S3:** Type III ANOVA results (Satterthwaite's method) for the additional parameters involved in the amortization time and RCC calculations. All models included a tree ID as random intercept. **Table S4:** Type III ANOVA results (Satterthwaite's method) for the additional parameters of the relative starch C cost calculation. All models included a tree ID as random intercept.

## Data Availability

Previously published data is available at https://doi.org/10.6084/m9.figshare.21952826 and https://doi.org/10.6084/m9.figshare.23559837, remaining data is available at https://doi.org/10.6084/m9.figshare.30963520.

## References

[pce70413-bib-0001] Bachofen, C. , P. D'Odorico , and N. Buchmann . 2020. “Light and VPD Gradients Drive Foliar Nitrogen Partitioning and Photosynthesis in the Canopy of European Beech and Silver Fir.” Oecologia 192: 323–339. 31901980 10.1007/s00442-019-04583-x

[pce70413-bib-0002] Baruch, Z. , R. R. Pattison , and G. Goldstein . 2000. “Responses to Light and Water Availability of Four Invasive Melastomataceae in the Hawaiian Islands.” International Journal of Plant Sciences 161: 107–118. 10648200 10.1086/314233

[pce70413-bib-0003] Bates, D. , M. Maechler , B. Bolker , and S. Walker . 2015. “Fitting Linear Mixed‐Effects Models Using Lme4.” Journal of Statistical Software 1: 1–48.

[pce70413-bib-0004] Boardman, N. K. 1977. “Comparative Photosynthesis of Sun and Shade Plants.” Annual Review of Plant Physiology 28: 355–377.

[pce70413-bib-0005] Burgess, S. S. , J. Pittermann , and T. E. Dawson . 2006. “Hydraulic Efficiency and Safety of Branch Xylem Increases With Height in Sequoia Sempervirens (D. Don) Crowns.” Plant, Cell and Environment 29: 229–239. 10.1111/j.1365-3040.2005.01415.x 17080638

[pce70413-bib-0006] Cavatte, P. C. , N. F. Rodríguez‐López , S. C. V. Martins , M. S. Mattos , L. M. V. P. Sanglard , and F. M. DaMatta . 2012. “Functional Analysis of the Relative Growth Rate, Chemical Composition, Construction and Maintenance Costs, and the Payback Time of Coffea Arabica L. Leaves in Response to Light and Water Availability.” Journal of Experimental Botany 63: 3071–3082. 22378951 10.1093/jxb/ers027 PMC3350923

[pce70413-bib-0007] Chabot, B. F. , and D. J. Hicks . 1982. “The Ecology of Leaf Life Spans.” Annual Review of Ecology and Systematics 13: 229–259.

[pce70413-bib-0008] Chen, J. M. , G. Mo , J. Pisek , et al. 2012. “Effects of Foliage Clumping on the Estimation of Global Terrestrial Gross Primary Productivity.” Global Biogeochemical Cycles 26.

[pce70413-bib-0009] Chung, H.‐H. , and R. L. Barnes . 1977. “Photosynthate Allocation Inpinustaeda. I. Substrate Requirements for Synthesis of Shoot Biomass.” Canadian Journal of Forest Research 7: 106–111.

[pce70413-bib-0010] Close, D. , C. McArthur , S. Paterson , H. Fitzgerald , A. Walsh , and T. Kincade . 2003. “Photoinhibition: A Link Between Effects of the Environment on Eucalypt Leaf Chemistry and Herbivory.” Ecology 84: 2952–2966.

[pce70413-bib-0011] Corner, E. J. H. 1949. “The Durian Theory or the Origin of the Modern Tree.” Annals of Botany 13: 367–414.

[pce70413-bib-0012] Daudet, F. A. , X. Le Roux , H. Sinoquet , and B. Adam . 1999. “Wind Speed and Leaf Boundary Layer Conductance Variation Within Tree Crown.” Agricultural and Forest Meteorology 97: 171–185.

[pce70413-bib-0013] Durand, M. , Z. R. Stangl , Y. Salmon , A. J. Burgess , E. H. Murchie , and T. M. Robson . 2022. “Sunflecks in the Upper Canopy: Dynamics of Light‐Use Efficiency in Sun and Shade Leaves of Fagus Sylvatica.” New Phytologist 235: 1365–1378. 35569099 10.1111/nph.18222 PMC9543657

[pce70413-bib-0014] Eliáš, P. , I. Kratochvílová , D. Janouš , M. Marek , and E. Masarovičová . 1989. “Stand Microclimate and Physiological Activity of Tree Leaves in an Oak‐Hornbeam Forest.” Trees 3: 227–233.

[pce70413-bib-0015] Ellsworth, D. S. , and P. B. Reich . 1993. “Canopy Structure and Vertical Patterns of Photosynthesis and Related Leaf Traits in a Deciduous Forest.” Oecologia 96: 169–178. 28313412 10.1007/BF00317729

[pce70413-bib-0016] Evans, J. R. , and H. Poorter . 2001. “Photosynthetic Acclimation of Plants to Growth Irradiance: The Relative Importance of Specific Leaf Area and Nitrogen Partitioning in Maximizing Carbon Gain.” Plant, Cell and Environment 24: 755–767.

[pce70413-bib-0017] Gressler, E. , S. Jochner , R. M. Capdevielle‐Vargas , L. P. C. Morellato , and A. Menzel . 2015. “Vertical Variation in Autumn Leaf Phenology of Fagus Sylvatica L. in Southern Germany.” Agricultural and Forest Meteorology 201: 176–186.

[pce70413-bib-0018] Griffin, K. L. 1994. “Calorimetric Estimates of Construction Cost and Their Use in Ecological Studies.” Functional Ecology 8: 551.

[pce70413-bib-0019] Gutschick, V. P. , and F. W. Wiegel . 1988. “Optimizing the Canopy Photosynthetic Rate by Patterns of Investment in Specific Leaf Mass.” American Naturalist 132: 67–86.

[pce70413-bib-0020] Hagemeier, M. , and C. Leuschner . 2019. “Functional Crown Architecture of Five Temperate Broadleaf Tree Species: Vertical Gradients in Leaf Morphology, Leaf Angle, and Leaf Area Density.” Forests 10: 265.

[pce70413-bib-0021] Hertel, C. , M. Leuchner , and A. Menzel . 2011. “Vertical Variability of Spectral Ratios in a Mature Mixed Forest Stand.” Agricultural and Forest Meteorology 151: 1096–1105.

[pce70413-bib-0022] Hoch, G. , A. Richter , and C. Körner . 2003. “Non‐Structural Carbon Compounds in Temperate Forest Trees.” Plant, Cell and Environment 26: 1067–1081.

[pce70413-bib-0023] Hollinger, D. Y. 1996. “Optimality and Nitrogen Allocation in a Tree Canopy.” Tree Physiology 16: 627–634. 14871700 10.1093/treephys/16.7.627

[pce70413-bib-0024] Jifon, J. L. , and J. P. Syvertsen . 2003. “Moderate Shade Can Increase Net Gas Exchange and Reduce Photoinhibition in Citrus Leaves.” Tree Physiology 23: 119–127. 12533306 10.1093/treephys/23.2.119

[pce70413-bib-0025] Keel, S. G. , and C. Schadel . 2010. “Expanding Leaves of Mature Deciduous Forest Trees Rapidly Become Autotrophic.” Tree Physiology 30: 1253–1259. 20688879 10.1093/treephys/tpq071

[pce70413-bib-0026] Kikuzawa, K. , and M. J. Lechowicz . 2011. Ecology of Leaf Longevity. Springer.

[pce70413-bib-0027] Klein, T. , Y. Vitasse , and G. Hoch . 2016. “Coordination Between Growth, Phenology and Carbon Storage in Three Coexisting Deciduous Tree Species in a Temperate Forest.” Tree Physiology 36: 847–855. 27126226 10.1093/treephys/tpw030

[pce70413-bib-0028] Koike, T. , M. Kitao , Y. Maruyama , S. Mori , and T. T. Lei . 2001. “Leaf Morphology and Photosynthetic Adjustments Among Deciduous Broad‐Leaved Trees Within the Vertical Canopy Profile.” Tree Physiology 21: 951–958. 11498342 10.1093/treephys/21.12-13.951

[pce70413-bib-0029] Koike, T. , S. Kitaoka , T. Ichie , T. T. Lei , and M. Kitao . 2004. “Photosynthetic Characteristics of Mixed Deciduous‐broadleaf Forests From Leaf to Stand.” In Global Environmental Change in the Ocean and on Land, edited by M. Shiyomi , 453–472. Terrapub.

[pce70413-bib-0030] Kothari, S. , J. Urgoiti , C. Messier , W. S. Keeton , and A. Paquette . 2025. “Self‐Pruning in Tree Crowns is Influenced by Functional Strategies and Neighbourhood Interactions.” Functional Ecology 39: 2234–2250.

[pce70413-bib-0031] Kramer, K. 1995. “Modelling Comparison to Evaluate the Importance of Phenology for the Effects of Climate Change on Growth of Temperate‐Zone Deciduous Trees.” Climate Research 5: 119–130.

[pce70413-bib-0032] Kuznetsova, A. , P. B. Brockhoff , and R. H. B. Christensen . 2017. “LmerTest Package: Tests in Linear Mixed Effects Models.” Journal of Statistical Software 82: 1–26.

[pce70413-bib-0033] Lacointe, A. , E. Deleens , T. Ameglio , et al. 2004. “Testing the Branch Autonomy Theory: A 13C/14C Double‐Labelling Experiment on Differentially Shaded Branches.” Plant, Cell and Environment 27: 1159–1168.

[pce70413-bib-0034] Landhäusser, S. M. 2011. “Aspen Shoots are Carbon Autonomous During Bud Break.” Trees 25: 531–536.

[pce70413-bib-0035] Larcher, W. 2003. Physiological Plant Ecology. Springer.

[pce70413-bib-0036] Law, B. E. , E. Falge , L. Gu , et al. 2002. “Environmental Controls over Carbon Dioxide and Water Vapor Exchange of Terrestrial Vegetation.” Agricultural and Forest Meteorology 113: 97–120.

[pce70413-bib-0037] Lenth, R. V. 2022. emmeans: Estimated Marginal Means, aka Least‐Squares Means

[pce70413-bib-0038] Leuschner, C. , and I. C. Meier . 2018. “The Ecology of Central European Tree Species: Trait Spectra, Functional Trade‐Offs, and Ecological Classification of Adult Trees.” Perspectives in Plant Ecology, Evolution and Systematics 33: 89–103.

[pce70413-bib-0039] Lewis, J. D. , R. B. McKane , D. T. Tingey , and P. A. Beedlow . 2000. “Vertical Gradients in Photosynthetic Light Response Within an Old‐Growth Douglas‐Fir and Western Hemlock Canopy.” Tree Physiology 20: 447–456. 12651440 10.1093/treephys/20.7.447

[pce70413-bib-0040] Li, J. , T.‐M. Ou‐Lee , R. Raba , R. G. Amundson , and R. L. Last . 1993. “Arabidopsis Flavonoid Mutants are Hypersensitive to UV‐B Irradiation.” Plant Cell 5: 171. 12271060 10.1105/tpc.5.2.171 PMC160260

[pce70413-bib-0041] Long, F. L. 1934. “Application of Calorimetric Methods to Ecological Research.” Plant Physiology 9: 323–337. 16652883 10.1104/pp.9.2.323 PMC439065

[pce70413-bib-0042] Lusk, C. H. , M. Jimnez‐Castillo , and N. Salazar‐Ortega . 2007. “Evidence That Branches of Evergreen Angiosperm and Coniferous Trees Differ in Hydraulic Conductance but Not in Huber Values.” Botany 85: 141–147.

[pce70413-bib-0043] Möhl, P. , E. Hiltbrunner , and C. Körner . 2020. “Halving Sunlight Reveals no Carbon Limitation of Aboveground Biomass Production in Alpine Grassland.” Global Change Biology 26: 1857–1872. 31799736 10.1111/gcb.14949

[pce70413-bib-0044] Mooney, H. A. , and S. L. Gulmon . 1982. “Constraints on Leaf Structure and Function in Reference to Herbivory.” BioScience 32: 198–206.

[pce70413-bib-0045] Niinemets, Ü. 1997. “Role of Foliar Nitrogen in Light Harvesting and Shade Tolerance of Four Temperate Deciduous Woody Species.” Functional Ecology 11: 518–531.

[pce70413-bib-0046] Niinemets, Ü. 1998. “Adjustment of Foliage Structure and Function to a Canopy Light Gradient in Two Co‐Existing Deciduous Trees. Variability in Leaf Inclination Angles in Relation to Petiole Morphology.” Trees 12: 446–451.

[pce70413-bib-0047] Niinemets, Ü. 1999. “Energy Requirement for Foliage Formation Is Not Constant Along Canopy Light Gradients in Temperate Deciduous Trees.” New Phytologist 141: 459–470.

[pce70413-bib-0048] Niinemets, U. , O. Kull , and J. D. Tenhunen . 1998. “An Analysis of Light Effects on Foliar Morphology, Physiology, and Light Interception in Temperate Deciduous Woody Species of Contrasting Shade Tolerance.” Tree Physiology 18: 681–696. 12651418 10.1093/treephys/18.10.681

[pce70413-bib-0049] Niinemets, Ü. , A. Portsmuth , and M. Tobias . 2006. “Leaf Size Modifies Support Biomass Distribution Among Stems, Petioles and Mid‐Ribs in Temperate Plants.” New Phytologist 171: 91–104. 16771985 10.1111/j.1469-8137.2006.01741.x

[pce70413-bib-0050] Niinemets, Ü. , E. Sonninen , and M. Tobias . 2004. “Canopy Gradients in Leaf Intercellular CO2 Mole Fractions Revisited: Interactions Between Leaf Irradiance and Water Stress Need Consideration.” Plant, Cell and Environment 27: 569–583.

[pce70413-bib-0051] Pickup, M. , M. Westoby , and A. Basden . 2005. “Dry Mass Costs of Deploying Leaf Area in Relation to Leaf Size.” Functional Ecology 19: 88–97.

[pce70413-bib-0052] Poorter, H. 1994. “Construction Costs and Payback Time of Biomass: A Whole Plant Perspective.” In A Whole Plant Perspective on Carbon‐nitrogen Interactions, edited by J. Roy and E. Garnier , 111–127. SPB Academic Publishing.

[pce70413-bib-0053] Poorter, H. , Ü. Niinemets , L. Poorter , I. J. Wright , and R. Villar . 2009. “Causes and Consequences of Variation in Leaf Mass Per Area (LMA): a Meta‐Analysis.” New Phytologist 182: 565–588. 19434804 10.1111/j.1469-8137.2009.02830.x

[pce70413-bib-0054] Poorter, H. , S. Pepin , T. Rijkers , Y. de Jong , J. R. Evans , and C. Körner . 2006. “Construction Costs, Chemical Composition and Payback Time of High‐ and Low‐Irradiance Leaves.” Journal of Experimental Botany 57: 355–371. 16303828 10.1093/jxb/erj002

[pce70413-bib-0055] Poorter, H. , and R. Villar . 1997. “The Fate of Acquired Carbon in Plants: Chemical Composition and Construction Costs.” In Plant Resource Allocation, edited by F. A. Bazzaz and J. Grace , 39–72. Academic Press.

[pce70413-bib-0056] R Core Team . 2025. *R: A Language and Environment for Statistical Computing*. R Foundation for Statistical Computing, Vienna, Austria.

[pce70413-bib-0057] Schadel, C. , A. Blochl , A. Richter , and G. Hoch . 2009. “Short‐Term Dynamics of Nonstructural Carbohydrates and Hemicelluloses in Young Branches of Temperate Forest Trees During Bud Break.” Tree Physiology 29: 901–911. 19457884 10.1093/treephys/tpp034

[pce70413-bib-0058] Schoonmaker, A. L. , R. M. Hillabrand , V. J. Lieffers , P. S. Chow , and S. M. Landhäusser . 2021. “Seasonal Dynamics of Non‐Structural Carbon Pools and Their Relationship to Growth in Two Boreal Conifer Tree Species.” Tree Physiology 41: 1563–1582. 33554258 10.1093/treephys/tpab013

[pce70413-bib-0059] Sellin, A. , and P. Kupper . 2006. “Spatial Variation in Sapwood Area to Leaf Area Ratio and Specific Leaf Area Within a Crown of Silver Birch.” Trees 20: 311–319.

[pce70413-bib-0060] Sims, D. A. , and R. W. Pearcy . 1991. “Photosynthesis and Respiration in Alocasia Macrorrhiza Following Transfers to High and Low Light.” Oecologia 86: 447–453. 28312935 10.1007/BF00317615

[pce70413-bib-0061] Sprugel, D. G. 2002. “When Branch Autonomy Fails: Milton's Law of Resource Availability and Allocation.” Tree Physiology 22: 1119–1124. 12414371 10.1093/treephys/22.15-16.1119

[pce70413-bib-0062] Sprugel, D. G. , T. M. Hinckley , and W. Schaap . 1991. “The Theory and Practice of Branch Autonomy.” Annual Review of Ecology and Systematics 22: 309–334.

[pce70413-bib-0063] Steubing, L. , C. Ramirez , and M. Alberdi . 1979. “Artenzusammensetzung, Lichtgenuß Und Energiegehalt Der Krautschicht Des Valdivianischen Regenwaldes Bei St. Martin.” Vegetatio 39: 25–33.

[pce70413-bib-0064] Sun, J. , M. Wang , M. Lyu , et al. 2019. “Stem Diameter (and Not Length) Limits Twig Leaf Biomass.” Frontiers in Plant Science 10: 185. 30846996 10.3389/fpls.2019.00185 PMC6393343

[pce70413-bib-0065] Vertregt, N. , and F. W. T. Penning de Vries . 1987. “A Rapid Method for Determining the Efficiency of Biosynthesis of Plant Biomass.” Journal of Theoretical Biology 128: 109–119.

[pce70413-bib-0066] Volpe, G. , R. Lo Bianco , and M. Rieger . 2008. “Carbon Autonomy of Peach Shoots Determined by 13C‐Photoassimilate Transport.” Tree Physiology 28: 1805–1812. 19193563 10.1093/treephys/28.12.1805

[pce70413-bib-0067] Way, D. A. , and R. W. Pearcy . 2012. “Sunflecks in Trees and Forests: From Photosynthetic Physiology to Global Change Biology.” Tree Physiology 32: 1066–1081. 22887371 10.1093/treephys/tps064

[pce70413-bib-0068] Webb, W. L. , M. Newton , and D. Starr . 1974. “Carbon Dioxide Exchange of Alnus Rubra. A Mathematical Model.” Oecologia 17: 281–291. 28308943 10.1007/BF00345747

[pce70413-bib-0069] Weber, R. , A. Gessler , and G. Hoch . 2019. “High Carbon Storage in Carbon‐Limited Trees.” New Phytologist 222: 171–182. 30451299 10.1111/nph.15599

[pce70413-bib-0070] Westoby, M. , and I. J. Wright . 2003. “The Leaf Size – Twig Size Spectrum and its Relationship to Other Important Spectra of Variation Among Species.” Oecologia 135: 621–628. 16228258 10.1007/s00442-003-1231-6

[pce70413-bib-0071] White, P. S. 1983. “Corner's Rules in Eastern Deciduous Trees: Allometry and Its Implications for the Adaptive Architecture of Trees.” Bulletin of the Torrey Botanical Club 110: 203.

[pce70413-bib-0072] Wilhelm, C. , and D. Selmar . 2011. “Energy Dissipation is an Essential Mechanism to Sustain the Viability of Plants: The Physiological Limits of Improved Photosynthesis.” Journal of Plant Physiology 168: 79–87. 20800930 10.1016/j.jplph.2010.07.012

[pce70413-bib-0073] Williams, K. , C. B. Field , and H. A. Mooney . 1989. “Relationships Among Leaf Construction Cost, Leaf Longevity, and Light Environment in Rain‐Forest Plants of the Genus Piper.” American Naturalist 133: 198–211.

[pce70413-bib-0074] Williams, K. , F. Percival , J. Merino , and H. A. Merino . 1987. “Estimation of Tissue Construction Cost From Heat of Combustion and Organic Nitrogen Content.” Plant, Cell and Environment 10: 725–734.

[pce70413-bib-0075] Witowski, J. 1997. “Gas Exchange of the Lowest Branches of Young Scots Pine: A Cost‐Benefit Analysis of Seasonal Branch Carbon Budget.” Tree Physiology 17: 757–765. 14759885 10.1093/treephys/17.12.757

[pce70413-bib-0076] Yang, D. , G. Li , and S. Sun . 2009. “The Effects of Leaf Size, Leaf Habit, and Leaf Form on Leaf/Stem Relationships in Plant Twigs of Temperate Woody Species.” Journal of Vegetation Science 20: 359–366.

[pce70413-bib-0077] Yang, D. , K. J. Niklas , S. Xiang , and S. Sun . 2010. “Size‐Dependent Leaf Area Ratio in Plant Twigs: Implication for Leaf Size Optimization.” Annals of Botany 105: 71–77. 19864268 10.1093/aob/mcp262 PMC2794065

[pce70413-bib-0078] Yang, X.‐D. , E.‐R. Yan , S. X. Chang , X.‐H. Wang , Y.‐T. Zhao , and Q.‐R. Shi . 2014. “Twig–Leaf Size Relationships in Woody Plants Vary Intraspecifically Along a Soil Moisture Gradient.” Acta Oecologica 60: 17–25.

[pce70413-bib-0079] Yoshimura, K. 2013. “Influences of Phenological Differences on Leaf‐Level Carbon Budget Between the Upper and Lower Crown of Lyonia Ovalifolia.” Botany 91: 25–33.

[pce70413-bib-0080] Zach, A. , B. Schuldt , S. Brix , V. Horna , H. Culmsee , and C. Leuschner . 2010. “Vessel Diameter and Xylem Hydraulic Conductivity Increase With Tree Height in Tropical Rainforest Trees in Sulawesi, Indonesia.” Flora ‐ Morphology, Distribution, Functional Ecology of Plants 205: 506–512.

[pce70413-bib-0081] Zahnd, C. , M. Arend , A. Kahmen , and G. Hoch . 2023. “Microclimatic Gradients Cause Phenological Variations Within Temperate Tree Canopies in Autumn but Not in Spring.” Agricultural and Forest Meteorology 331: 109340.

[pce70413-bib-0082] Zahnd, C. , M. Zehnder , M. Arend , A. Kahmen , and G. Hoch . 2024. “Uniform Carbon Reserve Dynamics Along the Vertical Light Gradient in Mature Tree Crowns.” Tree Physiology 44: 232–245. 38198739 10.1093/treephys/tpae005 PMC11898625

[pce70413-bib-0083] Zhu, S.‐D. , R.‐H. Li , J. Song , et al. 2016. “Different Leaf Cost–Benefit Strategies of Ferns Distributed in Contrasting Light Habitats of Sub‐Tropical Forests.” Annals of Botany 117: 497–506. 26684751 10.1093/aob/mcv179 PMC4765538

[pce70413-bib-0084] Zuur, A. F. , E. N. Ieno , N. J. Walker , A. A. Saveliev , and G. M. Smith . 2009. Mixed Effects Models and Extensions in Ecology With R. Springer.

