# Supplementary material for: Similar Relative Carbon Costs for Construction and Storage of Sun and Shade Branches in Mature Temperate Trees
Source: Plant Cell Environ. 2026 Mar 4;49(6):3351–62. doi: 10.1111/pce.70413 (PMC13136552; doi:10.1111/pce.70413)
Supplement: Supplementary file 1 — Fig. S1: Relative carbon costs of current‐year branches when using different temperature corrections for photosynthesis and leaf respiration. Fig. S2: Seasonal C uptake in upper (plain) and lower (hashed) branches. Fig. S3: Fitted parameters from the photosynthetic light response curves in upper (plain) and lower (hashed) branches. Fig. S4: Specific leaf area (SLA; a) and leaf area to branch dry weight ratio (LA:BDW; b) in upper (plain) and lower (hashed) branches. Significances are based on post‐hoc t tests (* P < 0.05). Fig. S5: Twig (a) and foliage (b) tissue C concentration in upper (light grey) and lower (dark grey) branches. Significances are based on post‐hoc t tests (* P < 0.05). Fig. S6: Parameters used for the calculation of relative starch build‐up costs in upper (plain) and lower (hashed) branches. Table S1: Full type III ANOVA results (Satterthwaite's method), completing main text Table 1. Table S2: Type II ANOVA results for those parameters involved in the amortization time and RCC calculations for which the N was not sufficient to include a tree ID as random effect (Per‐area C uptake and fitted LRC parameters). Table S3: Type III ANOVA results (Satterthwaite's method) for the additional parameters involved in the amortization time and RCC calculations. All models included a tree ID as random intercept. Table S4: Type III ANOVA results (Satterthwaite's method) for the additional parameters of the relative starch C cost calculation. All models included a tree ID as random intercept. [file PCE-49-3351-s001.docx]

**Supplementary Material for “Similar relative carbon costs for construction and storage of sun and shade branches in mature temperate trees”**

Cedric Zahnd, Miro Zehnder, Ansgar Kahmen, Günter Hoch

**Table S1:** Full type III ANOVA results (Satterthwaite’s method), completing main text Table 1.

| Response | Variable | Sum Sq | Mean Sq. | Num. df | Den. df | F | *P* |
| --- | --- | --- | --- | --- | --- | --- | --- |
| Leaf amort. time | Crown position | 0.27 | 0.27 | 1 | 46 | 4.43 | 0.041 * |
| (log) | Species | 82.16 | 10.27 | 8 | 46 | 170.60 | < 0.001 *** |
|  | Crown position: Species | 3.60 | 0.45 | 8 | 46 | 7.47 | < 0.001 *** |
| Total amort. time | Crown position | 0.10 | 0.10 | 1 | 23 | 2.05 | 0.165 ^ns^ |
| (log) | Species | 79.68 | 9.96 | 8 | 23 | 213.26 | < 0.001 *** |
|  | Crown position: Species | 1.90 | 0.23 | 8 | 23 | 5.08 | 0.001 ** |
| Leaf RCC | Crown position | 0.26 | 0.26 | 1 | 23 | 9.67 | 0.005 ** |
| (log) | Species | 0.68 | 0.08 | 8 | 23 | 3.13 | 0.015 * |
|  | Crown position: Species | 0.84 | 0.10 | 8 | 23 | 3.85 | 0.005 ** |
| Twig RCC | Crown position | 0.27 | 0.26 | 1 | 23 | 1.74 | 0.200 ^ns^ |
| (log) | Species | 3.97 | 0.50 | 8 | 23 | 3.20 | 0.014 * |
|  | Crown position: Species | 1.78 | 0.22 | 8 | 23 | 1.44 | 0.234 ^ns^ |
| Total RCC | Crown position | 0.31 | 0.31 | 1 | 23 | 8.90 | 0.007 ** |
| (log) | Species | 0.84 | 0.11 | 8 | 23 | 3.04 | 0.018 * |
|  | Crown position: Species | 0.64 | 0.08 | 8 | 23 | 2.31 | 0.056 ^ns^ |
| Rel. starch cost | Crown position | 0.65 | 0.65 | 1 | 22 | 2.61 | 0.121 ^ns^ |
| (log) | Species | 66.55 | 8.32 | 8 | 22 | 33.57 | < 0.001 *** |
|  | Crown position: Species | 1.75 | 0.22 | 8 | 22 | 0.88 | 0.548 ^ns^ |

*** *P* ≤ 0.001; ** *P* ≤ 0.01; * *P* ≤ 0.05; ns *P* > 0.05

**Table S2:** Type II ANOVA results for those parameters involved in the amortization time and RCC calculations for which the N was not sufficient to include a tree ID as random effect (Per-area C uptake and fitted LRC parameters). These results refer to parameters presented in Figs. S1 and S2.

| Response | Variable | Sum Sq | df | F | *P* |
| --- | --- | --- | --- | --- | --- |
| C uptake | Crown position | 649284 | 1 | 88.74 | < 0.001 *** |
|  | Species | 1018720 | 8 | 17.41 | < 0.001 *** |
|  | Crown position: Species | 141470 | 8 | 2.42 | 0.025 * |
|  | Residuals | 424324 | 58 |  |  |
| P_gmax_ | Crown position | 72.47 | 1 | 10.54 | 0.004 ** |
|  | Species | 240.93 | 8 | 4.38 | 0.004 ** |
|  | Crown position: Species | 44.11 | 8 | 0.80 | 0.609 ^ns^ |
|  | Residuals | 123.79 | 18 |  |  |
| R_d_ | Crown position | 2.17 | 1 | 15.39 | < 0.001 *** |
|  | Species | 3.71 | 8 | 3.30 | 0.017 * |
|  | Crown position: Species | 0.20 | 8 | 0.17 | 0.992 ^ns^ |
|  | Residuals | 2.53 | 18 |  |  |
| Φ_Ι0_ | Crown position | 0.001 | 1 | 13.11 | 0.002 ** |
|  | Species | 0.008 | 8 | 10.23 | < 0.001 *** |
|  | Crown position: Species | 0.001 | 8 | 1.24 | 0.332 ^ns^ |
|  | Residuals | 0.002 | 18 |  |  |

*** *P* ≤ 0.001; ** *P* ≤ 0.01; * *P* ≤ 0.05; ns *P* > 0.05

**Table S3:** Type III ANOVA results (Satterthwaite’s method) for the additional parameters involved in the amortization time and RCC calculations. All models included a tree ID as random intercept. These results refer to the parameters presented in Figs. S1, S3 and S4.

| Response | Variable | Sum Sq | Mean Sq. | Num. df | Den. df | F | *P* |
| --- | --- | --- | --- | --- | --- | --- | --- |
| Branch C uptake | Crown position | 8.11 | 8.11 | 1 | 23 | 40.89 | < 0.001 *** |
| (log) | Species | 30.12 | 3.76 | 8 | 23 | 18.99 | < 0.001 *** |
|  | Crown position: Species | 4.29 | 0.54 | 8 | 23 | 2.71 | 0.029 * |
| SLA | Crown position | 2.16 | 2.16 | 1 | 46 | 87.08 | < 0.001 *** |
| (log) | Species | 31.02 | 3.88 | 8 | 46 | 156.60 | < 0.001 *** |
|  | Crown position: Species | 0.24 | 0.03 | 8 | 46 | 1.20 | 0.318 ^ns^ |
| LA: BDW | Crown position | 2.31 | 2.31 | 1 | 23 | 14.85 | < 0.001 *** |
| (log) | Species | 20.47 | 2.56 | 8 | 23 | 16.44 | < 0.001 *** |
|  | Crown position: Species | 1.94 | 0.24 | 8 | 23 | 1.56 | 0.193 ^ns^ |
| Branch C conc. | Crown position | 0.04 | 0.04 | 1 | 23 | 0.08 | 0.783 ^ns^ |
|  | Species | 110.79 | 13.85 | 8 | 23 | 24.97 | < 0.001 *** |
|  | Crown position: Species | 1.45 | 0.18 | 8 | 23 | 0.33 | 0.947 ^ns^ |
| Foliage C conc. | Crown position | 9.68 | 9.68 | 1 | 23 | 20.54 | < 0.001 *** |
|  | Species | 47.17 | 5.90 | 8 | 23 | 12.52 | < 0.001 *** |
|  | Crown position: Species | 8.51 | 1.06 | 8 | 23 | 2.26 | 0.060 ^ns^ |

*** *P* ≤ 0.001; ** *P* ≤ 0.01; * *P* ≤ 0.05; ns *P* > 0.05

**Table S4:** Type III ANOVA results (Satterthwaite’s method) for the additional parameters of the relative starch C cost calculation. All models included a tree ID as random intercept. These results refer to the parameters presented in Fig. S5.

| Response | Variable | Sum Sq | Mean Sq. | Num. df | Den. df | F | *P* |
| --- | --- | --- | --- | --- | --- | --- | --- |
| Needle or branch | Crown position | 0.72 | 0.72 | 1 | 22 | 2.30 | 0.144 ^ns^ |
| dry weight | Species | 72.79 | 9.10 | 8 | 22 | 29.13 | < 0.001 *** |
|  | Crown position: Species | 3.34 | 0.42 | 8 | 22 | 1.34 | 0.278 ^ns^ |
| Starch amplitude | Crown position | 0.02 | 0.02 | 1 | 22 | 0.39 | 0.537 ^ns^ |
|  | Species | 0.88 | 0.11 | 8 | 22 | 2.06 | 0.086 ^ns^ |
|  | Crown position: Species | 1.39 | 0.17 | 8 | 22 | 3.25 | 0.013 * |
| Total C in starch | Crown position | 0.49 | 0.49 | 1 | 44 | 1.18 | 0.283 ^ns^ |
|  | Species | 102.61 | 12.83 | 8 | 44 | 30.72 | < 0.001 *** |
|  | Crown position: Species | 5.09 | 0.64 | 8 | 44 | 1.52 | 0.177 ^ns^ |
| Time for starch | Crown position | 112.85 | 112.85 | 1 | 22 | 0.83 | 0.372 ^ns^ |
| accumulation | Species | 2338.64 | 292.33 | 8 | 22 | 2.16 | 0.074 ^ns^ |
|  | Crown position: Species | 2048.07 | 256.01 | 8 | 22 | 1.89 | 0.114 ^ns^ |

*** *P* ≤ 0.001; ** *P* ≤ 0.01; * *P* ≤ 0.05; ns *P* > 0.05


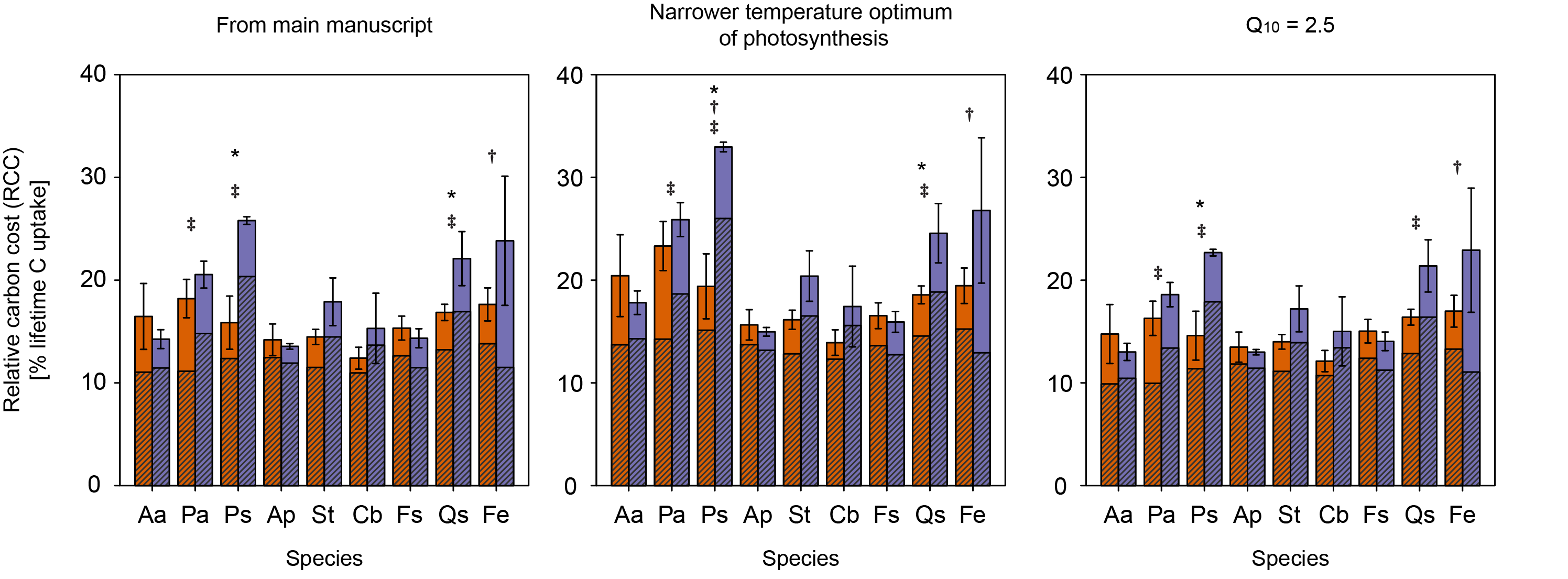


Fig. S1: Relative carbon costs of current-year branches when using different temperature corrections for photosynthesis and leaf respiration. Left panel is the same as Fig 1b in the main manuscript. In the middle panel, a narrower temperature optimum for photosynthesis was used (Optimum at 15-25°C, with linear declines reaching zero at 0°C and 40°C). In the left panel, a Q10 of 2.5 was used (Zheng et al., 2023). Each panel shows percentage of lifetime branch C uptake invested in leaf (hashed) and twig (wood + bark; plain) biomass in the upper (orange) and lower (purple) crown areas of the nine species. Standard error bars refer to the total values (leaf + twig). Species are shown on the x-axis (Aa = *Abies*, Pa = *Picea*, Ps = *Pinus*, Ap = *Acer*, St = *Sorbus*, Cb = *Carpinus*, Fs = *Fagus*, Qs = *Quercus*, Fe = *Fraxinus*). Significant differences between crown positions are indicated with different symbols for leaves (**‡**), twigs (**†**) or total (*****). Significances are based on post-hoc t tests (*P* < 0.05).

Reference:

Zheng, D.M., Wang, X., Liu, Q., Sun, Y.R., Ma, W.T., Li, L., Yang, Z., Tcherkez, G., Adams, M.A., Yang, Y. and Gong, X.Y. (2024), Temperature responses of leaf respiration in light and darkness are similar and modulated by leaf development. New Phytol, 241: 1435-1446.

**Fig. S2:** Seasonal C uptake in upper (plain) and lower (hashed) branches. (a) C uptake per m^2^ leaf area summed over the six summer months. (b) C uptake per branch (i.e. scaled to branch leaf area) over a full year (conifers) or the growing season (50 % bud break to 50 % leaf coloration; deciduous species). Significances are based on post-hoc t tests (* *P* < 0.05). Not the different y axis for *Fraxinus* in (b).

**Fig. S3:** Fitted parameters from the photosynthetic light response curves in upper (plain) and lower (hashed) branches. (a) P_gmax_, (b) R_d_ and (c) ${}_{(I_{0})}$. Since only two curves per species and crown position were measured, we did not test crown position differences for each species individually.

**Fig. S4:** Specific leaf area (SLA; a) and leaf area to branch dry weight ratio (LA:BDW; b) in upper (plain) and lower (hashed) branches. Significances are based on post-hoc t tests (* *P* < 0.05).

**Fig. S5:** Twig (a) and foliage (b) tissue C concentration in upper (light grey) and lower (dark grey) branches. Significances are based on post-hoc t tests (* *P* < 0.05).

**Fig. S6:** Parameters used for the calculation of relative starch build-up costs in upper (plain) and lower (hashed) branches. (a) Bulk dry weight of the needles (conifers) or branches (wood + bark; broadleaved). (b) Amplitude of seasonal starch dynamics (seasonal maximum minus seasonal minimum, see main text Fig. 3a). (c) Total amount of C [g] in starch when the seasonal maximum tissue concentration is reached. (d) Time from seasonal minimum to seasonal maximum tissue starch concentration (see main text Fig. 3a). Significances are based on post-hoc t tests (* *P* < 0.05). Note the different y-axes for *Fraxinus* in a and c.
